# A Manual of Medical Diagnosis

**Published:** 1858-08

**Authors:** 


ART. IX.—*A Manual of Medical Diagnosis. Being an Analysis of the Signs and Symptoms of Disease.* By A. W. Barclay, M. D., Cantab, et Edin., Fellow of the Royal College of Physicians; Assistant Physician to St. George's Hospital, etc., etc. Philadelphia: Blanchard & Lea. 1858.

We have not the space to devote to a critical review of the work before us, the title of which indicates its scope and importance. Too much cannot be said of the importance of correct diagnosis, without which clinical observations are valueless. What, for example, is more satisfactory than the present state of our knowledge in regard to treatment of diseases of the chest, for the reason that we are enabled by means of physical exploration, to know with almost absolute certainty, the extent and nature of pathological changes in that region? This single example shows that both treatment and prognosis are the more satisfactory in any class of diseases, as the means of diagnosis become more reliable. This truth is too evident to be discussed. If a book, then, will aid the student or practitioner a whit in the diagnosis of disease, it will always receive favor, at least from a certain class of the profession.

The scope of Dr. Barclay's work is so great, to be treated of in the space he has allowed himself, that we were prepared to find a few of the diagnostic signs of disease hastily and imperfectly considered. These omissions are fewer, however, than we could have anticipated, and on those subjects which are treated of more in detail, he advances sound and well established views. On the whole, we think it is a work well calculated to improve the element of diagnosis, in our study of disease, presenting many admirable suggestions which have naturally occurred to one of the attainments and large opportunities of Dr. Barclay.

The work has been republished in a neat octavo volume of 400 pages, in the elegant style which characterizes all the publications of Messrs. Blanchard & Lea.

